# Fault Diagnosis Method for Space Fluid Loop Systems Based on Improved Evidence Theory

**DOI:** 10.3390/e26050427

**Published:** 2024-05-16

**Authors:** Yue Liu, Zhenxiang Li, Lu Zhang, Hongyong Fu

**Affiliations:** 1 Key Laboratory of Space Utilization, Technology and Engineering Center for Space Utilization, Chinese Academy of Sciences, Beijing 100094, China; liuyue22@csu.ac.cn (Y.L.); lizhenxiang@csu.ac.cn (Z.L.); zhanglu@csu.ac.cn (L.Z.); 2 School of Aeronautics and Astronautics, University of Chinese Academy of Sciences, Beijing 100049, China

**Keywords:** fluid loop system for space applications, fault diagnosis, D-S evidence theory, Gaussian distribution, information fusion

## Abstract

Addressing the challenges posed by the complexity of the structure and the multitude of sensor types installed in space application fluid loop systems, this paper proposes a fault diagnosis method based on an improved D-S evidence theory. The method first employs the Gaussian affiliation function to convert the information acquired by sensors into BPA functions. Subsequently, it utilizes a pignistic probability transformation to convert the multiple subset focal elements into single subset focal elements. Finally, it comprehensively evaluates the credibility and uncertainty factors between evidences, introducing Bray–Curtis dissimilarity and belief entropy to achieve the fusion of conflicting evidence. The proposed method is initially validated on the classic Iris dataset, demonstrating its reliability. Furthermore, when applied to fault diagnosis in space application fluid circuit loop pumps, the results indicate that the method can effectively fuse multiple sensors and accurately identify faults.

## 1. Introduction

With the comprehensive completion of the Chinese Space Station in 2022, the station has entered a phase of application and development lasting more than ten years. During this phase, astronauts will reside continuously on the Space Station, utilizing the currently equipped space application system payloads to conduct nearly a thousand scientific research and application projects across multiple professional fields. Additionally, large-scale space science experiments and technological trials will be conducted, including research in space life sciences and human physiology, microgravity physics, astronomy, and earth sciences, as well as space new technologies and applications, aiming to promote the comprehensive development of China’s space science, technology, and applications [1,2,3,4,5,6].

The fluid loop system for space applications serves as a crucial mechanism within a space station for controlling and regulating the temperature of application system payloads. It primarily comprises a fluid loop host and key payload elements such as single-unit cold plate branches and thermal control drawer branches for scientific experiment cabinets. By employing a variety of active and passive thermal control measures, this system fulfills two primary functions. Firstly, it collects the heat generated by scientific payload equipment and associated support systems, transporting this thermal energy through the system’s fluid loop to the space station’s thermal control system. Ultimately, the heat is dissipated into the external environment via the station’s external radiator through radiative heat exchange. Secondly, through the operation of internal pumps and valve adjustments, the system maintains the temperatures of scientific payload equipment and support systems within permissible ranges, ensuring the normal operation and functionality of space application payloads.

The thermal control loop of the space station, serving as the thermal management system for space station application systems, holds significant importance for the success or failure of space scientific experiments and even the safety of spacecraft and astronauts [7]. The complex structure of the thermal control loop system, along with its diverse types of state-characteristic parameters, presents challenges. Among the core components of the thermal control fluid loop system, the circulation pump plays a critical role by providing pressure to the working medium. Any cessation of the circulation pump’s operation results in the stagnation of the working medium within the circulation pipeline, leading to a loss of temperature control capability within the fluid loop. Hence, there exists an urgent demand for enhanced fault diagnosis capabilities concerning the circulation pump within the thermal control loop. In the field of fault detection, compared to the limitations of single sensors, multi-sensor data fusion techniques [8,9,10,11,12,13,14] comprehensively consider information collected from multiple sensors. By analyzing the correlated decisions made by different sensors, comprehensive and reliable information can be obtained to accurately diagnose equipment faults. Therefore, it has been favored by experts and scholars both domestically and internationally. A related adaptive weighting method is proposed in reference [15], utilizing 1D-CNN for feature extraction, feature layer fusion, and fault classification of motor heterogeneous sensor information to achieve motor fault diagnosis. Reference [16] proposes a MICN, which processes signals from the same or different types of sensors, performs data fusion, and is used for bearing fault diagnosis. A multi-sensor data fusion method based on the D-S evidence theory is proposed in reference [17] for diagnosing faults in railway tracks.

In space application fluid circuit systems, temperature sensors, pressure sensors, and flow sensors are distributed in key areas, enabling real-time monitoring of temperature, pressure, and flow of applied fluid circuits. Through multi-sensor information fusion technology, equipment condition monitoring and fault diagnosis are achieved. As one of the methods of multi-sensor data fusion, the D-S evidence theory can effectively describe and express uncertain information without prior probabilities, making it widely applied in fault diagnosis [18,19,20,21,22,23,24], state assessment [25,26,27], and classification [28,29]. However, the evidence theory tends to fail when fusing conflicting evidence [30], and numerous studies have proposed improvements. Reference [31] proposes a conflict evidence fusion method based on evidence averaging weighting, but it assigns the same weight to each piece of evidence without considering their correlation, leading to poor accuracy of fusion results. Reference [32] proposes an evidence fusion method based on Mahalanobis distance; however, the calculation process of Mahalanobis distance is complex and not suitable for handling large-scale data. Reference [33] proposes a conflict evidence fusion method based on Jousselme distance, but Jousselme distance is influenced by the dispersion of evidence’s basic belief assignment functions, resulting in contradictory results when measuring evidence conflicts. Therefore, this paper proposes a conflict evidence fusion method based on Bray–Curtis dissimilarity and belief entropy [34], which weights evidence based on both the degree of evidence conflict and the amount of information, fully considering factors such as evidence credibility and uncertainty, thus better handling evidence conflicts and quickly and effectively identifying faults.

Furthermore, when reasoning based on the D-S evidence theory, the representation of uncertain information is a crucial issue to address. Specifically, converting the measured information from sensors into Basic Probability Assignment (BPA) functions poses a significant challenge in the practical application of the DS evidence theory. Currently, there is no universal rule for constructing BPA, and methods for their construction are mainly determined based on specific circumstances. Scholars have proposed various BPA generation methods from different perspectives. One approach, proposed in reference [35], is based on clustering principles, utilizing the K-means method to construct a model for generating basic probability assignments and subsequently determining the BPA function based on this model. Another method, presented in reference [36], utilizes the distance between triangular membership functions and test data to obtain BPA functions, defining a distance formula between two triangular membership functions. Additionally, reference [37] suggests a method for obtaining BPA functions based on normal distributions, where the evidence’s normal distribution model is established using training data and the relationship between test data and the normal distribution model is used to obtain the evidence’s BPA function. In a different approach, reference [38] proposes a BPA acquisition method based on the Adaboost algorithm, where multiple strong classifiers are generated for each attribute model using training data to determine the BPA of a single subset of focal elements. Lastly, reference [39] introduces a method to obtain basic probability assignments by constructing a BP neural network, leveraging the powerful self-learning and nonlinear mapping capabilities of BP neural networks to normalize output values and derive basic probability assignments.

Therefore, this paper proposes a fault detection method for spatially applied fluid circuits based on an improved D-S evidence theory. The method initially transforms sensor-acquired parameters into BPA functions using Gaussian affiliation functions. Subsequently, the BPA functions of multi-subset focal elements are converted into those of single-subset focal elements through pignistic probabilistic functions. Additionally, Bray–Curtis dissimilarity and belief entropy are employed to weigh and adjust the acquired BPA functions, effectively mitigating the influence of conflicting evidence. Finally, the fusion and identification of evidence are executed.

## 2. Preliminaries

**Definition** **1.** *The Frame of Discernment: In the D-S evidence theory [40,41,42,43], the totality of the research object is called identification frame* Θ*, and the elements in* Θ *are mutually exclusive.* Θ *= {*$A_{1},\;A_{2},\;\cdots ,A_{i}$*} denotes the set of all possible events, in which denotes the set of all possible events, in which* $A_{i}$ *is a subset of the identification frame* Θ*.*$2^{\Theta }$ *denotes the set consisting of all subsets.*

(1) $$2^{\Theta }=\left\{\emptyset ,\;\left\{A_{1}\right\},\;\cdots ,\left\{A_{n}\right\},\;\left\{A_{1},A_{2}\right\},\;\cdots ,\;\left\{A_{1},A_{2},A_{3}\right\},\;\cdots ,\;\Theta \right\}$$

*Here,*∅ *signifies the empty set,*$\left\{A_{1},\;A_{2}\right\}$ *represents*$\left\{A_{1}\cap A_{2}\right\}$.

**Definition** **2.** *Basic Probability Assignment: For any proposition* A *in* $2^{\Theta }$*,* *define the mapping* m *:* $2^{\Theta }\to [0,\;1]$ *to be a BPA function,* m *satisfying the following conditions*.

(2) $$\;\left\{\begin{array}{c} m\left(\emptyset \right)=0\\ 0\leq m\left(A\right)\leq 1\\ \sum_{A\subseteq 2^{\Theta }}m\left(A\right)=1 \end{array}\right.$$

m(A) *is the BPA function of proposition A, also known as the mass function. Proposition A is said to be a focal element if* m(A)>0*. When the focal elements are all singleton sets, such a focal element is called single-subset focal element evidence. Correspondingly, when the focal elements are multi-subsets, it is denoted as multi-subset focal element evidence*.

**Definition** **3.** *D-S Theory Synthesis Rules: For the identification frame* Θ*, there are two independent sources of evidence, and the BPA functions are* $m_{1}$ *and* $m_{2}$*. The combination rule for the D-S theory of evidence is follows:*

(3) $$m\left(A\right)=m_{1}\left(A_{i}\right)⊕m_{2}\left(A_{j}\right)=\left\{\begin{array}{c} 0,\;A=\emptyset \\ \frac{\sum_{A_{i}\cap A_{j}=A}m_{1}\left(A_{i}\right)m_{2}\left(A_{j}\right)}{1-k},A\neq \emptyset \end{array}\right.$$

 *where* $k=\sum_{A_{i}\cap A_{j}=\emptyset }m_{1}(A_{i})m_{2}(A_{j})$*, called the conflict coefficient, indicates the degree of conflict between two pieces of evidence, and* k∈[0, 1].

## 3. Materials and Methods

In order to gain a better understanding of fault diagnosis on space application fluid loop circulation pumps using an improved evidence theory, this section will introduce the method of BPA generation based on Gaussian affiliation function, pignistic transformation, and the evidence weighting method based on Bray–Curtis dissimilarity and belief entropy. These methodologies serve as theoretical foundations for subsequent applications in fluid loop systems.

### 3.1. Method for BPA Generation Based on Gaussian Affiliation Function

In practical applications, the sensor measurement environment undergoes real-time changes, leading to a degree of fuzziness in the measurement data. Therefore, the fuzzy set affiliation function is chosen to construct the BPA function. The Gaussian distribution offers several advantages, such as stability, symmetry, universality, and positive function value. Based on the Gaussian distribution, the Gaussian affiliation function directly reflects the relative probability that the sample belongs to the Gaussian function, thus retaining the many advantages of the Gaussian distribution. Consequently, this paper proposes a method based on the Gaussian affiliation function to obtain the BPA function.

In constructing the model utilizing Gaussian distributions [44], the raw dataset collected from multiple sensor measurements needs to be divided into training datasets and testing datasets. Within the training dataset, Gaussian models corresponding to different attributes’ data distributions can be obtained by computing the Gaussian affiliation function for each attribute. Subsequently, by matching the testing samples with the Gaussian models, the degree of match for each attribute can be determined, accurately depicting the sample’s characteristics across various attributes. Following this, normalization is applied to all match values, transforming them into BPA functions.

Assuming there are a total of n categories in the original dataset, forming the recognition framework θ, where $\theta =\left\{\;A_{1},\;A_{2},\cdots ,A_{n}\right\}$, each category contains samples with k attributes.

(1) Selection of training and testing samples:

Initially, extract m samples from the original dataset as training samples and use these samples to construct models based on the membership distribution of each attribute. Subsequently, designate the remaining data as testing samples, match them with the established models, and ultimately compute the samples’ BPA values.

(2) Construction of Gaussian models on each attribute:

During the data processing stage, Gaussian affiliation functions are employed to establish Gaussian models for the training samples across different attributes. This approach enables a more precise description of the range of attribute feature values, thereby enhancing the accuracy and reliability of the model. The Gaussian membership function expression is as follows:

(4) $$\;\;\;\;\;\;\;\;\;\mu \left(x\right):X\to \left[0,1\right],x\epsilon X$$

The mean $\bar{X_{ij}}$ and sample standard deviation $\sigma_{ij}$ of different categories i on different attributes j are calculated as follows:

(5) $$\;\;\;\;\bar{X_{ij}}=\frac{1}{q}\sum_{l=1}^{q}x_{ij}^{l}$$

(6) $$\;\;\;\;\;\;\sigma_{ij}=\sqrt{\frac{1}{q-1}\sum_{l=1}^{q}{\left(x_{ij}^{l}-\bar{X_{ıj}}\right)}^{2}}$$

where $i=1,2,\cdots n;j=1,2,\cdots ,k;x_{ij}^{l}$ denotes the value of the lth training sample in category i on the jth attribute.

The Gaussian-type membership function for category i on the jth attribute is given by:

(7) $$u_{i}^{j}\left(x\right)=e^{\frac{{-\left(x-\bar{X_{ij}}\right)}^{2}}{2{\sigma_{ij}}^{2}}}$$

where $-3\sigma_{ij}\leq x\leq 3\sigma_{ij},\;\;i=1,2,\cdots ,n,\;\;j=1,2,\cdots ,k$.

Thus, the membership distribution of samples across different attributes can be obtained.

(3) Matching testing samples with Gaussian models

By matching the samples in the test set with Gaussian models, the degree of similarity between them and different categories can be computed, thereby obtaining the matching values between samples and models. Normalize the matching values to obtain the BPA for each sample. Assuming Q is a proposition in the recognition framework, the formula for matching samples with Q is as follows:

(8) $$\;\;H\left(Q\leftarrow t\right)={\left.u_{Q}\left(X\right)\right|}_{x=t}$$

where t represents the value of the test sample on a specific attribute. The magnitude of H(Q←t) determines the degree of matching between the test sample and the Gaussian model, thus reflecting the accuracy of proposition  Q.

After matching, the required BPA can be obtained from the models. Arrange the values of the test sample in different categories in descending order as $H_{1},H_{2}$,$\cdots ,H_{n}$, then the calculation of its BPA is as follows:

(9) $$m_{1,2,\cdots ,n}=\frac{H_{n}}{\sum_{i=1}^{n}H_{i}}$$

### 3.2. Pignistic Probability Function

When dealing with the BPA function of multi-subset focal elements, the fusion outcome might indicate multiple focal elements, leading to less precise fault identification results. Hence, this paper employs the pignistic probabilistic transformation method [45] to quantify the multi-subset focal elements. This involves converting the BPA function of multi-subset focal elements into the probability distribution function of single-subset focal elements, thereby facilitating the ultimate fault identification process. The definition of the pignistic probabilistic transformation method is described as follows:

(10) $$B\mathrm{e}tPm\left(A_{i}\right)=\sum_{A_{i}\subseteq \Theta }\frac{\left|A_{i}\cap A\right|}{\left|A\right|}\frac{m\left(A\right)}{1-m\left(\emptyset \right)}$$

where A is the subset of the identification frame Θ, |·| denotes the number of focal elements contained in the subset.

### 3.3. Weight Determination Based on Credibility and Uncertainty

#### 3.3.1. Evidence Similarity Based on the Bray–Curtis Dissimilarity

The Bray–Curtis dissimilarity was proposed by J. Roger Bray and John T. Curtis in 1957 to measure the relative abundance of different species in ecology [46,47,48]. It serves to quantify the relative abundance among different species in ecology. This dissimilarity metric satisfies nonnegativity, symmetry, and normality criteria. As a nonparametric measure, it does not necessitate assumptions about probability distributions or parameterization, rendering it suitable for various types of uncertainty distributions. Furthermore, the Bray–Curtis dissimilarity is relatively simple to compute and is well-suited for large-scale data processing tasks, making it a viable option for calculating the degree of support between evidences.

Suppose there are two pieces of evidence in the identification frame Θ, the Bray–Curtis dissimilarity between the two pieces of evidence is:

(11) $$b\left(m_{i},m_{j}\right)=\frac{\sum_{k=1}^{M}\left|{BetP}_{m_{i}}\left(A_{k}\right)-{BetP}_{m_{j}}\left(A_{k}\right)\right|}{\sum_{k=1}^{M}{BetP}_{m_{i}}\left(A_{k}\right)+\sum_{k=1}^{M}{BetP}_{m_{j}}\left(A_{k}\right)}$$

where i,j=1,2,⋯,N.

The Bray–Curtis dissimilarity between the evidence $m_{i}$ and $m_{j}$ can be represented by the matrix B, which is an N-dimensional matrix:

(12) $$\;B=\left[\begin{array}{cc} \begin{array}{ccc} b_{11} & b_{12} & \ldots \\ b_{21} & b_{22} & \ldots \\ \vdots & \vdots & \ddots \end{array} & \begin{array}{ccc} b_{1j} & \ldots & b_{1N}\\ b_{2j} & \ldots & b_{2N}\\ \vdots & \ddots & \vdots \end{array}\\ \begin{array}{ccc} b_{i1} & b_{i2} & \ldots \\ \vdots & \vdots & \ddots \\ b_{N1} & b_{N2} & \ldots \end{array} & \begin{array}{ccc} b_{ij} & \ldots & b_{iN}\\ \vdots & \ddots & \vdots \\ b_{Nj} & \ldots & b_{NN} \end{array} \end{array}\right]$$

where $b_{ij}$ is $b(m_{i},m_{j})$, represents the Bray–Curtis dissimilarity between evidences $m_{i}$ and $m_{j}$. When i=j, $b_{ij}$ = 0. The range of Bray–Curtis dissimilarity is between 0 and 1, which is negatively correlated with the degree of similarity of the evidence. Thus, the system’s support for evidence is defined as follows:

(13) $$SUP\left(m_{i}\right)=\frac{1}{N-1}\sum_{j=1,j\neq i}^{N}\frac{1}{b_{ij}}$$

The weight of evidence $m_{i}$ can be obtained after normalization as ${WR\_B}_{\left(m_{i}\right)}$:

(14) $${WR\_B}_{\left(m_{i}\right)}=\frac{SUP\left(m_{i}\right)}{\sum_{j=1}^{N}SUP\left(m_{j}\right)}$$

where i=1,2,⋯,N.

#### 3.3.2. Evidence Uncertainty Based on Entropy

Shannon entropy, proposed by Shannon [49], is a classic method for measuring information uncertainty, commonly used to describe the amount of information contained in the states of a random variable. At the same time, Deng [50] introduced the concept of belief entropy as a universal improvement of Shannon entropy and applied it to the D-S evidence theory. A lower belief entropy indicates lower uncertainty and higher credibility of evidence, while a higher belief entropy signifies greater uncertainty and lower credibility of evidence. In this paper, belief entropy was introduced to measure the uncertainty and credibility of evidence. Hypothesis $m_{i}$ is a mass function defined in the identification frame Θ, the belief entropy corresponding to evidence is denoted as $H\left(m_{i}\right)$.

(15) $$H\left(m_{i}\right)=-\sum_{A_{n}\subseteq \Theta }m_{i}\left(A_{n}\right)\log_{2}\frac{m_{i}\left(A_{n}\right)}{2^{\left|A_{n}\right|}-1}$$

where $A_{n}(n=1,\;2,\;\cdots ,\;N)$ is a proposition in mass function $m_{i}$, and $\left|A_{n}\right|$ is the cardinality of $A_{n}$.

To prevent the assignment of zero weight to evidence $m_{i}$ in certain circumstances, the magnitude of evidence weight is determined by computing the exponential form of belief entropy.

(16) $$\;E_{i}(m_{i})=e^{H\left(m_{i}\right)}=e^{-\sum_{A_{n}\in \Theta }m_{i}\left(A_{n}\right)\log_{2}m_{i}\left(A_{n}\right)}$$

After normalization, the uncertainty of evidence $m_{i}$ is:

(17) $$\;{WR\_E}_{\left(m_{i}\right)}=\frac{{E_{i}}_{\left(m_{i}\right)}}{\sum_{i=1}^{N}{E_{i}}_{\left(m_{i}\right)}}$$

#### 3.3.3. Evidence Fusion Based on the Dempster Rule

By computing the Bray–Curtis dissimilarity and belief entropy between evidences, the information effect between evidences can be amplified. For evidence with higher credibility, higher weights are assigned. Therefore, by cascading the weighting coefficients based on Bray–Curtis dissimilarity and belief entropy, the weighted correction coefficients for evidence are determined as follows:

(18) $$\;{W_{i}}_{\left(m_{i}\right)}={WR\_B}_{\left(m_{i}\right)}\times {WR\_E}_{\left(m_{i}\right)}$$

The weighted correction coefficients ${W_{i}}_{\left(m_{i}\right)}$ are normalized to obtain evidence fusion coefficients ${W_{FUS}}_{\left(m_{i}\right)}$:

(19) $$\;{W_{FUS}}_{\left(m_{i}\right)}=\frac{{W_{i}}_{\left(m_{i}\right)}}{\sum_{i=1}^{N}{W_{i}}_{\left(m_{i}\right)}}$$

The mass function values of evidence $m_{i}$ are each assigned a corresponding weighted correction factor to obtain the corrected evidence $m_{i}^{\prime }$:

(20) $$\;m_{i}^{\prime }=\sum_{i=1}^{N}{W_{FUS}}_{\left(m_{i}\right)}\times m_{i}$$

where i=1,2,⋯,n.

N−1 fusion of $m_{i}^{\prime }$ by the Dempster rule produces evidence fusion results in the following:

(21) $$\;m_{FUS}={\left({\left({\left(m_{i}^{\prime }⊕m_{i}^{\prime }\right)}_{1}\cdots \right)}_{i}⊕m_{i}^{\prime }\right)}_{N-1}$$

### 3.4. The Proposed Fault Diagnosis Method

**Figure 1:**
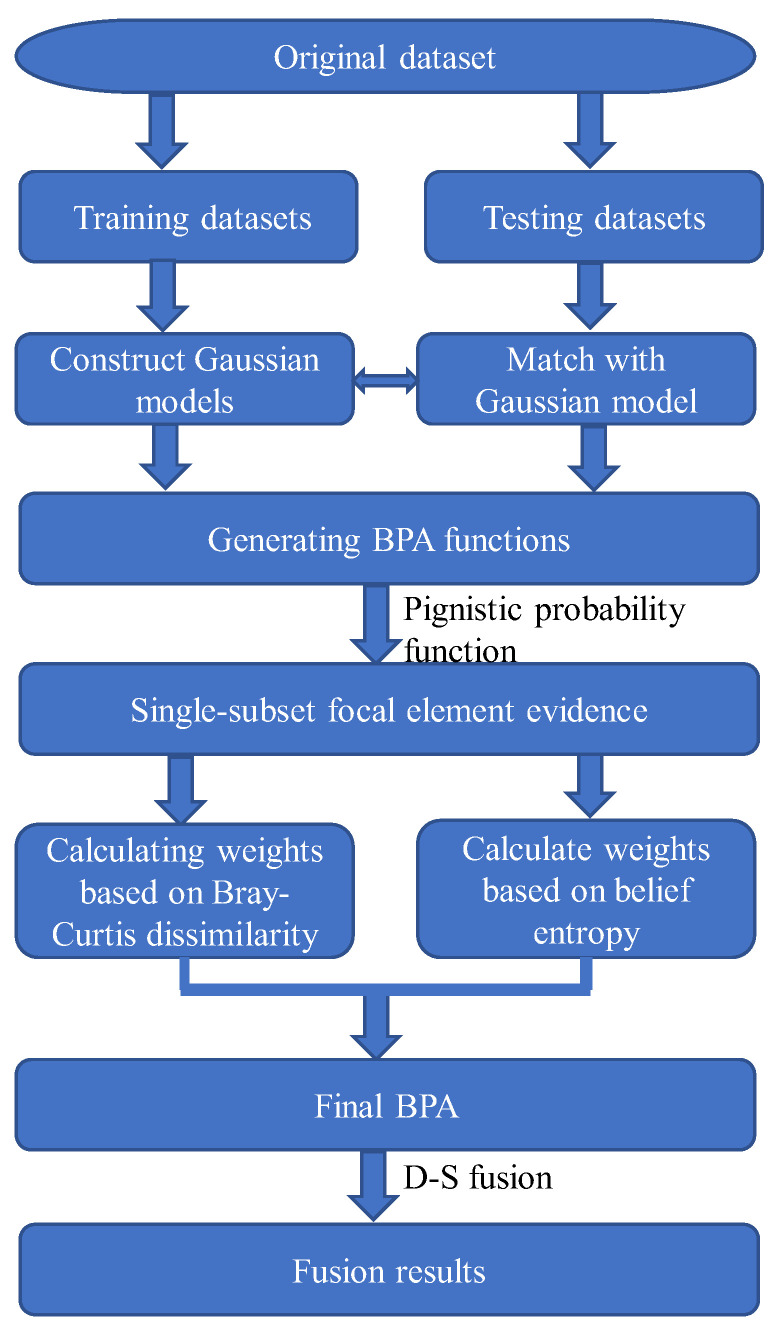
Flowchart of the proposed method.

The paper proposes a fault diagnosis method based on improved D-S evidence theory. Firstly, it introduces a BPA generation method based on the Gaussian affiliation function, leveraging fuzzy set theory and Gaussian distribution models, which exhibit good reliability and practicality, effectively transforming parameters obtained from sensors into BPA functions. Subsequently, it utilizes pignistic probability transformation to convert multi-subset focal elements into single-subset focal elements. Moreover, it fully considers factors of evidence credibility and uncertainty, presenting a conflict evidence fusion method based on Bray–Curtis dissimilarity and belief entropy. This method measures evidence from both dissimilarity and information content aspects, determining the final weighted correction coefficients for evidence and facilitating effective data fusion and ultimate decision-making. The detailed steps of this method include the following eight steps, with the method flowchart depicted in Figure 1.

(1) Partition the original dataset into training and testing datasets. Select a portion of the original dataset as training samples and utilize them for constructing Gaussian models.

(2) Compute the mean and standard deviation of the training samples, conduct Gaussian model construction, and obtain Gaussian models for each category across different attributes.

(3) Match the testing samples with Gaussian models to derive the corresponding BPA functions.

(4) Employ pignistic probability transformation to convert multi-subset focal elements into single-subset focal elements, facilitating accurate fault diagnosis outcomes.

(5) After obtaining the BPA for each attribute, compute the Bray–Curtis dissimilarity between evidences and determine the system’s support and weights for evidence.

(6) Calculate the belief entropy for each piece of evidence, as well as obtain their respective weights.

(7) Determine the weighted correction coefficients for each piece of evidence from the perspectives of evidence dissimilarity and uncertainty and conduct weighted correction on the evidence.

(8) Utilize the Dempster combination rule to fuse the corrected BPA functions and obtain the final fault diagnosis result.

## 4. Experiments

To validate the effectiveness of the proposed fault diagnosis method in this paper, two sets of cases were selected for validation and analysis. The first set of data is the publicly available Iris flower dataset from UCI [51], while the second set involves real-world applications in the space application fluid loop. Through these two case studies, the BPA function generation method based on Gaussian affiliation function and the conflict evidence fusion method based on Bray–Curtis dissimilarity and belief entropy are demonstrated in detail, validating the effectiveness of the proposed method in this paper.

### 4.1. Iris Data Set Classification

In this section, the classical dataset Iris dataset in the UCI Machine Learning Library is taken as an example, and the computational process of the BPA generation method based on the Gaussian model is given in detail, as well as the process of weighting and fusion of the evidence. The validity of the proposed method in this chapter is verified through the classification experiments on the Iris dataset.

The Iris dataset contains three categories of iris flowers: Setosa (S), Versicolor (E), and Virginica (V), which constitute a recognition framework θ, where θ={S, E, V}. Each category comprises 50 samples, totaling 150 samples. Each sample consists of four feature attributes: sepal length (SL/cm), sepal width (SW/cm), petal length (PL/cm), and petal width (PW/cm).

From each of the three iris categories (S, E, and V), 30 random samples are selected to form the training set, with the remaining 20 samples forming the test set. The mean and standard deviation of the 30 training samples on the four attributes SL, SW, PL, and PW are calculated, respectively, using Equations (5) and (6). The specific calculation results are presented in Table 1.

**Figure 2:**
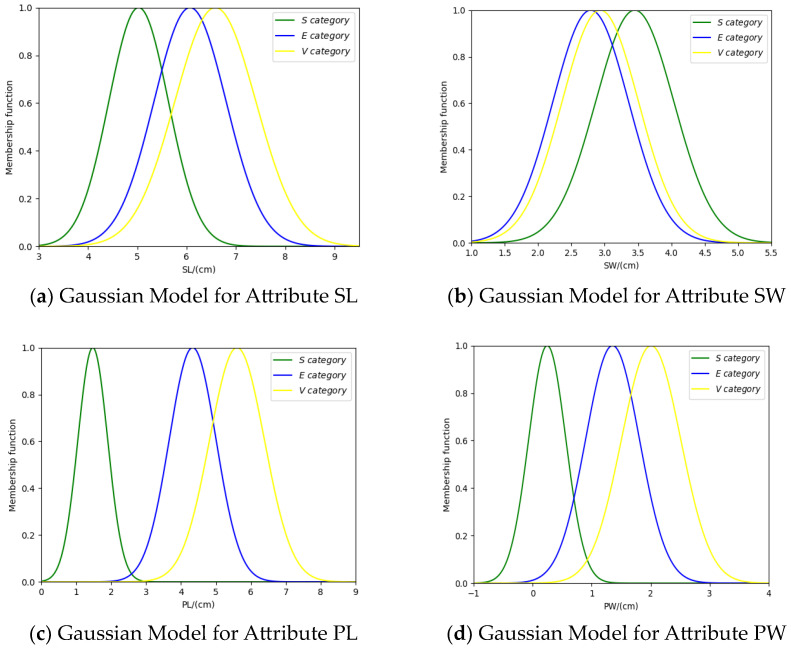
Gaussian models for four attributes.

Based on the mean and standard deviation, the Gaussian models are constructed for the training samples of each category on each attribute. The Gaussian models are shown in Figure 2 below.

For the attribute SL, the membership functions $u_{S}\left(x\right),\;\;u_{E}\left(x\right),\;\;u_{V}(x)$ represent the categories S, E, and V, respectively. The membership function $u_{SE}(x)$ represents that the attribute model can be classified as both category S and category E. The membership function $u_{SEV}(x)$ represents that the attribute model can be classified as category S, category E, and category V. Their mathematical expressions are as follows:

$$u_{SE}\left(x\right)=min\left[u_{S}\left(x\right),u_{E}(x)\right]$$

$$u_{SEV}\left(x\right)=min\left[u_{S}\left(x\right),u_{E}(x),u_{V}(x)\right]$$

By matching the testing samples with the Gaussian models corresponding to each category, we obtain the degree of match between the testing samples and each category. Then, by normalization, we obtain the BPA.

**Figure 3:**
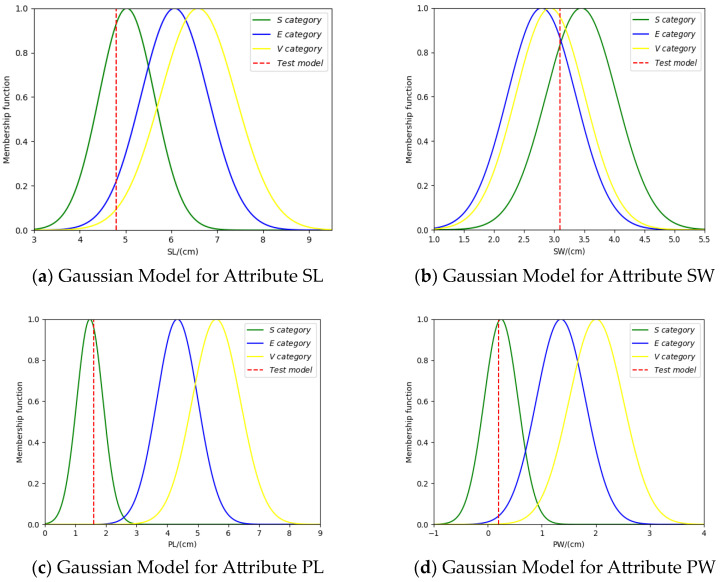
Matching degree between testing sample and Gaussian models.

Taking class S as an example, randomly select a sample $x=[x_{1},x_{2},x_{3},x_{4}]$ = [4.8, 3.1, 1.6, 0.2], from the test set, where $x_{1},x_{2},x_{3},x_{4}$ represent the feature values of this sample on the four attributes SL, SW, PL, and PW. By matching the sample x with the Gaussian models of each category, we obtain the degree of match between the sample x and each Gaussian model, as shown in Figure 3 below.

Finally, we obtained four sets of BPA values for the testing sample based on the four attributes SL, SW, PL, and PW. The specific numerical values are shown in Table 2 below.

The current BPA functions belong to high-conflict evidence with multiple subset focal elements. Directly fusing the BPA functions at this stage will yield incorrect identification information. Firstly, utilize pignistic probability transformation to convert the current BPA functions into single-subset focal elements. The transformation results are shown in Table 3 below.

The Bray–Curtis dissimilarity matrix is derived from Equations (11) and (12) as follows:

$$B=\left[\begin{array}{cc} \begin{array}{cc} 0 & 0.7549\\ 0.7549 & 0 \end{array} & \begin{array}{cc} 0.1397 & 0.1197\\ 0.8946 & 0.8746 \end{array}\\ \begin{array}{cc} 0.1397 & 0.8946\\ 0.1197 & 0.8746 \end{array} & \begin{array}{cc} 0 & 0.0200\\ 0.0200 & 0 \end{array} \end{array}\right]$$

Applying Equation (13), the evidence support based on the Bray–Curtis dissimilarity matrix is determined as follows:

$$SUP\left(m_{i}^{\prime }\right)=\left\{4.2093,0.8965,14.5690,14.8744\right\}$$

Utilizing Equation (14) for normalization, the support coefficients become as follows:

$${WR\_B}_{\left(m_{i}^{\prime }\right)}=\left\{0.1218,0.0259,0.4217,0.4305\right\}$$

Applying Equations (15) and (16), the entropy of each piece of evidence is calculated as follows:

$$E_{i}(m_{i}^{\prime })=\left\{1.9731,3.5674,1.0030,1.1543\right\}$$

Normalization of the evidence uncertainty coefficient according to Equation (17) yields the following:

$${WR\_E}_{\left(m_{i}^{\prime }\right)}=\left\{0.2563,0.4634,0.1303,0.1500\right\}$$

According to Equations (18) and (19), the normalized evidence weighted correction coefficient is calculated as follows:

$${W_{FUS}}_{\left(m_{i}^{\prime }\right)}=\left\{0.1918,0.0737,0.3376,0.3968\right\}$$

Applying Equation (20), a corresponding correction coefficient is assigned to each piece of evidence, resulting in the final mass function value:

$$m_{i}^{\prime }=\left\{0.8991,0.0496,0.0512\right\}$$

Evidence fusion is executed according to Equation (21), and the final fusion results are presented in Table 4. The fusion result successfully identifies the sample category as class S.

Testing on the remaining 59 training samples from classes S, E, and V yielded an overall recognition rate of 98.45%, confirming the effectiveness of the proposed method.

### 4.2. Application in Fault Diagnosis of Fluid Circuit Loop Pumps

In this section, the computational process of the BPA generation method based on the Gaussian affiliation function is given in detail by applying the telemetry data of fluid loop circulation pumps as an example, and the improved DS evidence theory method proposed in this paper is used for fault diagnosis, which verifies the practical application effect of the method proposed in this paper.

**Figure 4:**
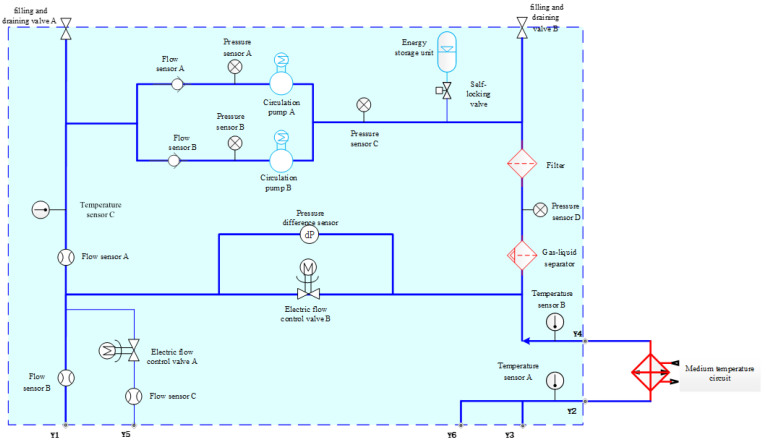
System configuration and sensor positioning.

The application fluid circuit system consists of essential components such as a circulation pump, measuring sensors, energy storage unit, filter, gas-liquid separator, self-locking valve, filling and draining valve, one-way valve, electric flow control valve, and so on. The system configuration and sensor positioning are illustrated in Figure 4 below.

The application fluid circuit loop pump serves as the core power equipment for space application fluid circuits. Its main functions include providing pressure to the working medium of the driving components, driving the medium circulation, and achieving self-sealing functionality. Once the circulation pump fails, the medium in the circulation pipeline ceases to flow, resulting in the loss of the fluid circuit’s temperature control capability, potentially causing localized temperatures to rise in some loads. A standby design is implemented for the circulation pump to guarantee system reliability.

The energy storage unit, as a stabilizing element within the system, ensuring the circulation pump operates within normal pressure parameters. The primary role of the filter is to eliminate impurities from the working medium, thereby enhancing the efficiency of the fluid circuit. At the same time, the gas-liquid separator is pivotal in isolating and expelling air bubbles entrapped within the circulating medium, thereby maintaining the normal operation of the application fluid circuit. Additionally, various types of valve components are strategically positioned throughout the main circuit of the fluid circuit system to ensure unhindered flow of the working medium, encompassing self-locking valves, two electric flow control valves (electric flow control valve A and electric flow control valve B), two check valves (check valve A and check valve B), and two injection discharge valves (injection discharge valve A and injection discharge valve B).

Various types of sensors are strategically deployed at critical junctures within the application fluid circuit system, including three temperature sensors (temperature sensor A, temperature sensor B, and temperature sensor C), three pressure sensors (pressure sensor A, pressure sensor B, and pressure sensor C), three flow sensors (flow sensor A, flow sensor B, and flow sensor C), and a differential pressure sensor. These sensors facilitate real-time measurement and display of the system’s temperature, pressure, flow rate, pressure differential, and liquid level within the accumulator. During normal operation of the application fluid circuit system, sensor readings remain within a dynamic range. Any deviation from this range indicates system malfunction, with the degree of fault directly correlated with the extent of deviation in sensor readings. Leveraging the embedded sensors within the system enables effective state monitoring.

Analyzing the telemetry parameters of the circulation pump and the monitoring parameters related to the health status of the circulation pump, an analysis is conducted on the sensor types and telemetry parameters of the circulation pump. Based on the layout of internal sensors in the circulation pump, four factors are selected to assess the likelihood of faults occurring: the rotational speed value of circulation pump A, the pressure value of pressure sensor A, the pressure value of pressure sensor C, and the level value of the energy storage tank gauge. From the perspective of evidence theory, the information obtained from each sensor can be regarded as evidence, and fault diagnosis based on multi-sensor information is essentially an evidence fusion problem. The corresponding relationship between fault modes and relevant monitoring parameters is presented in Table 5. The fault modes $A_{1}$, $A_{2}$ and $A_{3}$ are all random static faults.

Through fault simulation of the space application fluid circuit loop pump, 50 fault data samples are collected for each fault mode. Each fault mode records four feature attributes, namely the rotational speed value of circulation pump A (X1), the pressure value of pressure sensor A (X2), the pressure value of pressure sensor C (X3), and the level value of the energy storage tank gauge (X4).

From the three categories of fault data samples, $A_{1},\;\;A_{2},\;\;A_{3}$, 30 samples are randomly selected as training models, while the remaining 20 samples are used as testing samples for model validation.

**Figure 5:**
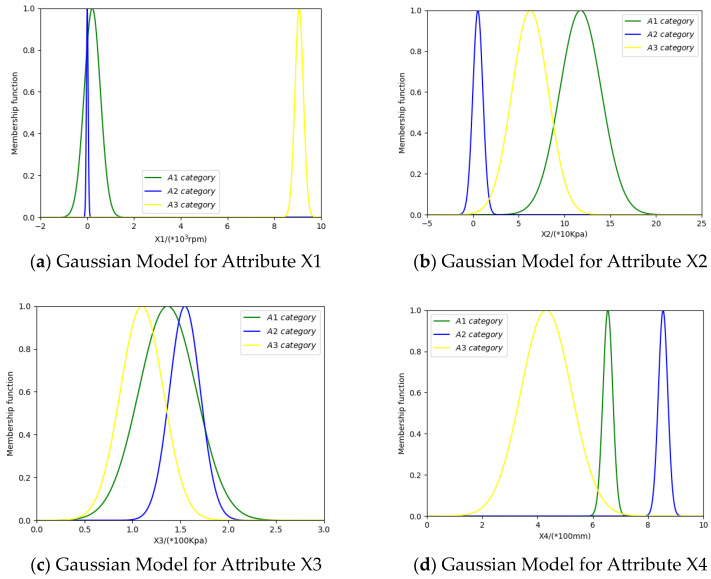
Gaussian models for four attributes.

The mean and standard deviation of the 30 training samples on attributes X1, X2, X3, and X4 are calculated, respectively, and then the Gaussian models for each category of training samples on each attribute are constructed. The Gaussian models corresponding to the four attributes are shown in Figure 5 below.

**Figure 6:**
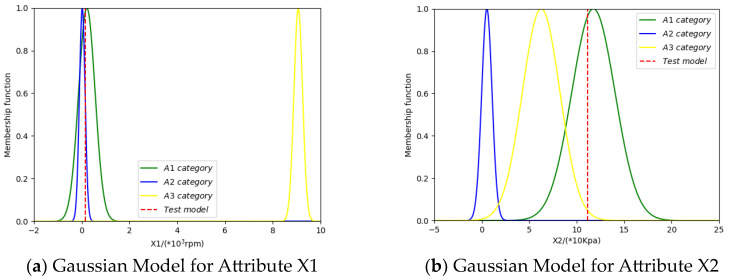

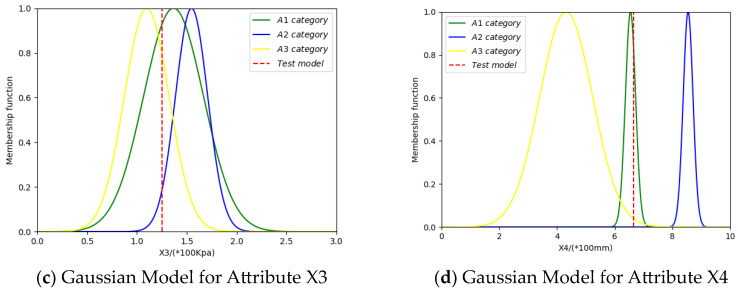
Matching degree between the testing sample and gaussian models.

Taking class $A_{1}$ as an example, a sample $x=[x_{1},x_{2},x_{3},x_{4}]$ = [0.5241, 11.1642, 1.2547, 6.6462], is randomly selected from the test set, where $x_{1},x_{2},x_{3},x_{4}$ denote the eigenvalues of this sample on four attributes, namely, X1, X2, X3 and X4. By matching the sample x with the Gaussian models of each category, we obtain the degree of match between the sample x and each Gaussian model, as shown in Figure 6 below.

Finally, we obtained four sets of BPA values for the testing sample based on the four attributes SL, SW, PL, and PW. The specific numerical values are shown in Table 6 below.

The current BPA function was converted to a single subset of focal elements using a pignistic probability transformation, and the converted BPA function is shown in Table 7 below.

The Bray–Curtis dissimilarity matrix is obtained by applying Equations (11) and (12) as follows:

$$B=\left[\begin{array}{cc} \begin{array}{cc} 0 & 0.1774\\ 0.1774 & 0 \end{array} & \begin{array}{cc} 0.2389 & 0.1768\\ 0.2472 & 0.0026 \end{array}\\ \begin{array}{cc} 0.2389 & 0.2472\\ 0.1768 & 0.0026 \end{array} & \begin{array}{cc} 0 & 0.2492\\ 0.2492 & 0 \end{array} \end{array}\right]$$

Applying Equation (13), the evidence support based on the Bray–Curtis dissimilarity matrix is determined as follows:

$$SUP\left(m_{i}^{\prime }\right)=\left\{3.8697,98.5744,3.0610,98.5711\right\}$$

Utilizing Equation (14) for normalization, the support coefficients become as follows:

$${WR\_B}_{\left(m_{i}^{\prime }\right)}=\left\{0.0190,0.4830,0.0150,0.4830\right\}$$

Applying Equations (15) and (16), the entropy of each piece of evidence is calculated as follows:

$$E_{i}(m_{i}^{\prime })=\left\{1.9628,1.1862,2.6974,1.1784\right\}$$

Normalization of the evidence uncertainty coefficient according to Equation (17) yields the following:

$${WR\_E}_{\left(m_{i}^{\prime }\right)}=\left\{0.2794,0.1689,0.3840,0.1677\right\}$$

According to Equations (18) and (19), the normalized evidence weighted correction coefficient is calculated as follows:

$${W_{FUS}}_{\left(m_{i}^{\prime }\right)}=\left\{0.0306,0.4698,0.0332,0.4664\right\}$$

Applying Equation (20), a corresponding correction coefficient is assigned to each piece of evidence, resulting in the final mass function value:

$$m_{i}^{\prime }=\left\{0.9627,0.0068,0.0305\right\}$$

Evidence fusion is executed according to Equation (21), and the final fusion results are presented in Table 8.

Testing on the remaining 59 training samples from classes $A_{1},\;\;A_{2}$ and $A_{3}$ yielded an overall recognition rate of 98%, validating the effectiveness of the method. This confirms the potential applicability of the method for practical fault detection in space application fluid circuit loop pumps.

## 5. Conclusions

This paper proposes a fault diagnosis method for space fluid circuit loop pumps based on an improved D-S evidence theory. Initially, Gaussian membership functions are employed to obtain the corresponding BPA functions for the evidence acquired from sensors, facilitating the quantitative representation of sensor signals. Subsequently, multi-subset focal element evidence is converted to single-subset focal element evidence using the pignistic probability function, enhancing the accuracy of fault identification. Furthermore, to address conflicting evidence fusion in D-S evidence theory, a conflict evidence fusion method based on Bray–Curtis dissimilarity, and belief entropy is introduced. This method comprehensively evaluates evidence similarity and information content, determining the credibility of evidence using Bray–Curtis dissimilarity and assessing evidence uncertainty using belief entropy. Weighting correction coefficients for evidence are then determined based on a comprehensive assessment of the credibility and uncertainty of the evidence. Finally, the fusion of corrected evidence using D-S evidence theory yields the ultimate fault diagnosis. The main contributions of this paper are as follows:

(1) Addressing the ambiguity of sensor signals in the practical working environment of spatially applied fluid circuit loop pumps by introducing Gaussian models to determine BPA functions for each attribute. This enables the quantitative representation of sensor signals and facilitates more accurate fault identification through the conversion of multi-subset focal element evidence to single-subset focal element evidence using the pignistic probability function.
(2) Proposing a conflict evidence fusion method based on Bray–Curtis dissimilarity and belief entropy for handling conflicting evidence in D-S evidence theory. This method integrates the assessment of evidence similarity and information content to determine evidence credibility and uncertainty, respectively. Weighting correction coefficients for evidence are then determined based on this comprehensive assessment, leading to the final fault diagnosis using D-S evidence theory.
(3) The fault diagnosis method for space fluid circuit loop pumps based on the improved D-S evidence theory effectively addresses the ambiguity of sensor signals and the conflict after signal interference in the equipment environment, thus aligning well with the actual operating conditions of spatially applied fluid circuit loop pumps and demonstrating strong robustness.

## Figures and Tables

**Table 1:** Mean value and standard deviation of the training samples.

Category	$\left(\bar{X_{SL},}\;\sigma_{SL}\right)$	$\left(\bar{X_{SW},}\;\sigma_{SW}\right)$	$\left(\bar{X_{PL},}\;\sigma_{PL}\right)$	$\left(\bar{X_{PW},}\;\sigma_{PW}\right)$
S	$\left(5.0267,\;0.3660\right)$	$\left(3.4500,\;0.3442\right)$	$\left(1.4733,\;0.1825\right)$	$\left(0.2467,\;0.0991\right)$
E	$\left(6.0700,\;0.5367\right)$	$\left(2.7900,\;0.3229\right)$	$\left(4.3333,\;0.4519\right)$	$\left(1.3533,\;0.2077\right)$
V	$\left(6.5833,\;0.6773\right)$	$\left(2.9333,\;0.3290\right)$	$\left(5.6033,\;0.6162\right)$	$\left(2.0067,\;0.2516\right)$

**Table 2:** BPA Functions for the testing sample on each attribute.

Category	$m\left(\left\{S\right\}\right)$	$m\left(\left\{E\right\}\right)$	$m\left(\left\{V\right\}\right)$	$m\left(\left\{S,E\right\}\right)$	$m\left(\left\{S,V\right\}\right)$	$m\left(\left\{E,V\right\}\right)$	$m\left(\left\{S,E,V\right\}\right)$
$m_{1}({BPA}_{SL})$	0.9320	0.0000	0.0000	0.2219	0.0000	0.0000	0.0950
$m_{2}({BPA}_{SW})$	0.0000	0.0000	0.9606	0.0000	0.0000	0.8596	0.8391
$m_{3}({BPA}_{PL})$	0.9569	0.0000	0.0000	0.0000	0.0000	0.0000	0.0003
$m_{4}({BPA}_{PW})$	0.9887	0.0000	0.0000	0.0412	0.0000	0.0000	0.0003

**Table 3:** BPA functions after pignistic probability transformation.

Category	$m\left(\left\{S\right\}\right)$	$m\left(\left\{E\right\}\right)$	$m\left(\left\{V\right\}\right)$
$m_{1}^{\prime }({BPA}_{SL})$	0.8601	0.1145	0.0254
$m_{2}^{\prime }({BPA}_{SW})$	0.1052	0.2668	0.6280
$m_{3}^{\prime }({BPA}_{PL})$	0.9998	0.0001	0.0001
$m_{4}^{\prime }({BPA}_{PW})$	0.9798	0.0201	0.0001

**Table 4:** Final fusion results.

Fusion Results	$m\left(\left\{S\right\}\right)$	$m\left(\left\{E\right\}\right)$	$m\left(\left\{V\right\}\right)$
$m_{1}^{\prime }⊕m_{2}^{\prime }$	0.9938	0.0030	0.0032
$m_{1}^{\prime }⊕m_{2}^{\prime }⊕m_{3}^{\prime }$	0.9996	0.0002	0.0002
$m_{1}^{\prime }⊕m_{2}^{\prime }⊕m_{3}^{\prime }⊕m_{4}^{\prime }$	1.0000	0.0000	0.0000

**Table 5:** Correspondence between circulation pump fault modes and relevant monitoring parameters.

Project Name	Fault Mode	Fault Diagnosis Method	Telemetry Available for Fault Diagnosis
Space Application Fluid Circuit Loop Pump	$A_{1}$ (Circulation Pump speed reduction)	Decrease in Circulation Pump Speed, decrease in Internal Pressure Circulation pump	A rotational speed value, Pressure sensor A pressure value, Pressure sensor C pressure value, Energy storage tank gauge value
	$A_{2}$ (Circulation Pump Shutdown)	Gradual decrease in circulation pump speed to zero, decrease in internal pressure	
	$A_{3}$ (Circulation Pump Leakage)	Decrease in circulation pump speed, decrease in internal pressure, decrease in system flow rate	

**Table 6:** BPA Functions for the testing sample on each attribute.

Category	$m\left(\left\{A_{1}\right\}\right)$	$m\left(\left\{A_{2}\right\}\right)$	$m\left(\left\{A_{3}\right\}\right)$	$m\left(\left\{A_{1},A_{2}\right\}\right)$	$m\left(\left\{A_{1},A_{3}\right\}\right)$	$m\left(\left\{A_{2},A_{3}\right\}\right)$	$m\left(\left\{A_{1},A_{2},A_{3}\right\}\right)$
$m_{1}({BPA}_{X1})$	0.978	0.0000	0.0000	0.538	0.0000	0.0000	0.0000
$m_{2}({BPA}_{X2})$	0.972	0.0000	0.0000	0.0000	0.052	0.0000	0.0000
$m_{3}({BPA}_{X3})$	0.932	0.0000	0.0000	0.0000	0.783	0.0000	0.1928
$m_{4}({BPA}_{X4})$	0.845	0.0000	0.0000	0.0000	0.0395	0.0000	0.0015

**Table 7:** BPA functions after pignistic probability transformation.

Category	$m\left(\left\{A_{1}\right\}\right)$	$m\left(\left\{A_{2}\right\}\right)$	$m\left(\left\{A_{3}\right\}\right)$
$m_{1}({BPA}_{X1})$	0.8226	0.1774	0.0000
$m_{2}({BPA}_{X2})$	0.9746	0.0000	0.0254
$m_{3}({BPA}_{X3})$	0.7274	0.0337	0.2389
$m_{4}({BPA}_{X4})$	0.9766	0.0006	0.0228

**Table 8:** Final fusion results.

Fusion Results	$m\left(\left\{A_{1}\right\}\right)$	$m\left(\left\{A_{2}\right\}\right)$	$m\left(\left\{A_{3}\right\}\right)$
$m_{1}^{\prime }⊕m_{2}^{\prime }$	0.9989	0.0001	0.0010
$m_{1}^{\prime }⊕m_{2}^{\prime }⊕m_{3}^{\prime }$	0.9999	0.0000	0.0001
$m_{1}^{\prime }⊕m_{2}^{\prime }⊕m_{3}^{\prime }⊕m_{4}^{\prime }$	1.0000	0.0000	0.0000

## Data Availability

Data are contained within the article.

## References

[1] Liu F., Wu S., Zheng W., Yuan Y., Tian Q., Fan P., Wu M., Zhang T., Yu L., Wang J. (2023). Research and Development of Cell Culture Devices Aboard the Chinese Space Station. Microgravity Sci. Technol..

[2] Rojas-Alva U., Jomaas G. (2022). A historical overview of experimental solid combustion research in microgravity. Acta Astronaut..

[3] Liu Q., Liu R. (2011). Influence of high-frequency vibration on the Rayleigh–Marangoni instability in a two-layer system. Phys. Fluids.

[4] Liu C.J., Yang X., Mao Y., Zhang X.X., Wu X.T., Wang S.H., Fan Y.B., Sun W.E. (2023). The alteration of advanced glycation end products and its potential role on bone loss under microgravity. Acta Astronaut..

[5] Xue T., Liang D., Pang W., Shen D., Niamat A., Liu J., Zhou J. (2022). Ignition and combustion of metal fuels under microgravity: A short review. FirePhysChem.

[6] Liu L.J., Fan Y.B., Wang S.H., Wu X.T., Yang X., Sun L.W. (2023). Enhanced osteogenic potential of periosteal stem cells maintained cortical bone relatively stable under microgravity. Acta Astronaut..

[7] Cao Z., Zhang J., Li Z., Zhao X., Guo D., Fu H. (2022). Study on Prognostics and Health Management of Fluid Loop System for Space Application. J. Phys. Conf. Ser..

[8] Sasiadek J.Z. (2002). Sensor fusion. Annu. Rev. Control..

[9] Fan X., Zuo M.J. (2006). Fault diagnosis of machines based on D–S evidence theory. Part 1: D–S evidence theory and its improvement. Pattern Recognit. Lett..

[10] Castanedo F. (2013). A review of data fusion techniques. Sci. World J..

[11] Kashinath S.A., Mostafa S.A., Mustapha A., Mahdin H., Lim D., Mahmoud M.A., Mohammed M.A., Al-Rimy B.A.S., Fudzee M.F.M., Yang T. (2021). Review of data fusion methods for real-time and multi-sensor traffic flow analysis. IEEE Access.

[12] Meng T., Jing X., Yan Z., Pedrycz W. (2020). A survey on machine learning for data fusion. Inf. Fusion.

[13] Yeong D.J., Velasco-Hernandez G., Barry J., Walsh J. (2021). Sensor and sensor fusion technology in autonomous vehicles: A review. Sensors.

[14] Li Y.F., Wang H., Sun M. (2023). ChatGPT-like large-scale foundation models for prognostics and health management: A survey and roadmaps. Reliab. Eng. Syst. Saf..

[15] Gu Y., Zhang Y., Yang M., Li C. (2023). Motor on-line fault diagnosis method research based on 1D-CNN and multi-sensor information. Appl. Sci..

[16] Wan S., Li T., Fang B., Yan K., Hong J., Li X. (2023). Bearing Fault Diagnosis Based on Multi-Sensor Information Coupling and Attentional Feature Fusion. IEEE Trans. Instrum. Meas..

[17] Oukhellou L., Debiolles A., Denœux T., Aknin P. (2010). Fault diagnosis in railway track circuits using Dempster–Shafer classifier fusion. Eng. Appl. Artif. Intell..

[18] Chen L., Diao L., Sang J. (2018). Weighted evidence combination rule based on evidence distance and uncertainty measure: An application in fault diagnosis. Math. Probl. Eng..

[19] Jiang W., Hu W., Xie C. (2017). A new engine fault diagnosis method based on multi-sensor data fusion. Applied Sciences.

[20] Jiang W., Xie C., Zhuang M., Shou Y., Tang Y. (2016). Sensor data fusion with z-numbers and its application in fault diagnosis. Sensors.

[21] Liu J., Chen A., Zhao N. (2018). An intelligent fault diagnosis method for bogie bearings of metro vehicles based on weighted improved DS evidence theory. Energies.

[22] Jiang D., Wang Z. (2023). Research on mechanical equipment fault diagnosis method based on deep learning and information fusion. Sensors.

[23] Huo Z., Martinez-Garcia M., Zhang Y., Shu L. (2021). A multisensor information fusion method for high-reliability fault diagnosis of rotating machinery. IEEE Trans. Instrum. Meas..

[24] Ghosh N., Saha S., Paul R. (2021). iDCR: Improved Dempster Combination Rule for multisensor fault diagnosis. Eng. Appl. Artif. Intell..

[25] Jia R.S., Liu C., Sun H.M., Yan X.H. (2015). A situation assessment method for rock burst based on multi-agent information fusion. Comput. Electr. Eng..

[26] Liu Z.G., Pan Q., Dezert J., Martin A. (2016). Adaptive imputation of missing values for incomplete pattern classification. Pattern Recognit..

[27] Khan M.N., Anwar S. (2019). Paradox elimination in Dempster–Shafer combination rule with novel entropy function: Application in decision-level multi-sensor fusion. Sensors.

[28] Belmahdi F., Lazri M., Ouallouche F., Labadi K., Absi R., Ameur S. (2023). Application of Dempster-Shafer theory for optimization of precipitation classification and estimation results from remote sensing data using machine learning. Remote Sens. Appl. Soc. Environ..

[29] Hamid R.A., Albahri A.S., Albahri O.S., Zaidan A.A. (2022). Dempster–Shafer theory for classification and hybridised models of multi-criteria decision analysis for prioritisation: A telemedicine framework for patients with heart diseases. J. Ambient. Intell. Humaniz. Comput..

[30] Zadeh L.A. (1984). Review of a mathematical theory of evidence. AI Mag..

[31] Murphy C.K. (2000). Combining belief functions when evidence conflicts. Decis. Support Syst..

[32] Yong D., Shi W., Zhu Z., Liu Q. (2004). Combining belief functions based on distance of evidence. Decis. Support Syst..

[33] Lin Y., Wang C., Ma C., Dou Z., Ma X. (2016). A new combination method for multisensor conflict information. J. Supercomput..

[34] Liu Y., Zou T., Fu H. (2024). A Conflict Evidence Fusion Method Based on Bray–Curtis Dissimilarity and the Belief Entropy. Symmetry.

[35] Fei L., Xia J., Feng Y., Liu L. (2019). A novel method to determine basic probability assignment in Dempster–Shafer theory and its application in multi-sensor information fusion. Int. J. Distrib. Sens. Netw..

[36] Numbers U.F. (2023). Determination of Basic Belief Assignment Using Fuzzy Numbers. Adv. Appl. DSmT Inf. Fusion.

[37] Xu P., Deng Y., Su X., Mahadevan S. (2013). A new method to determine basic probability assignment from training data. Knowl. Based Syst..

[38] Fu W., Yu S., Wang X. (2021). A novel method to determine basic probability assignment based on AdaBoost and its application in classification. Entropy.

[39] Zhang Z., Qinghai Y. (2018). Target recognition method based on BP neural networks and improved DS evidence theory. Comput. Appl. Softw..

[40] Dempster A.P. (1967). Upper and Lower Probabilities Induce by a Multiplicand Mapping. Ann. Math. Stat..

[41] Glenn S. (1976). A Mathematical Theory of Evidence.

[42] Pal N.R., Bezdek J.C., Hemasinha R. (1992). Uncertainty measures for evidential reasoning I: A review. Int. J. Approx. Reason..

[43] George T., Pal N.R. (1996). Quantification of conflict in Dempster-Shafer framework: A new approach. Int. J. Gen. Syst..

[44] Barnard G.A. (1949). Statistical inference. J. R. Stat. Society. Ser. B (Methodol.).

[45] Smets P., Kennes R. (2008). The transferable belief model. Class. Work. Dempster-Shafer Theory Belief Funct..

[46] Bray J.R., Curtis J.T. (1957). An ordination of the upland forest communities of southern Wisconsin. Ecol. Monogr..

[47] Ricotta C., Podani J. (2017). On some properties of the Bray-Curtis dissimilarity and their ecological meaning. Ecol. Complex..

[48] Ricotta C., Pavoine S. (2022). A new parametric measure of functional dissimilarity: Bridging the gap between the Bray-Curtis dissimilarity and the Euclidean distance. Ecol. Model..

[49] Shannon C.E. (2001). A mathematical theory of communication. ACM Sigmobile Mob. Comput. Commun. Rev..

[50] Deng Y. Deng Entropy: A Generalized Shannon Entropy to Measure Uncertainty. https://fs.unm.edu/DengEntropyAGeneralized.pdf.

[51] Fisher R.A. (1936). The use of multiple measurements in taxonomic problems. Ann. Eugen..

